# Single-Molecular Förster Resonance Energy Transfer Measurement on Structures and Interactions of Biomolecules

**DOI:** 10.3390/mi12050492

**Published:** 2021-04-27

**Authors:** Yi Qiao, Yuhan Luo, Naiyun Long, Yi Xing, Jing Tu

**Affiliations:** 1 State Key Laboratory of Bioelectronics, School of Biological Science and Medical Engineering, Southeast University, Nanjing 210096, China; yiqiao@seu.edu.cn (Y.Q.); 220202105@seu.edu.cn (Y.L.); 220191899@seu.edu.cn (N.L.); 2 Institute of Child and Adolescent Health, School of Public Health, Peking University, Beijing 100191, China; xingyang@bjmu.edu.cn

**Keywords:** FRET, smFRET, biomolecule, nucleic acid structure, protein

## Abstract

Single-molecule Förster resonance energy transfer (smFRET) inherits the strategy of measurement from the effective “spectroscopic ruler” FRET and can be utilized to observe molecular behaviors with relatively high throughput at nanometer scale. The simplicity in principle and configuration of smFRET make it easy to apply and couple with other technologies to comprehensively understand single-molecule dynamics in various application scenarios. Despite its widespread application, smFRET is continuously developing and novel studies based on the advanced platforms have been done. Here, we summarize some representative examples of smFRET research of recent years to exhibit the versatility and note typical strategies to further improve the performance of smFRET measurement on different biomolecules.

## 1. Introduction

**Figure 1:**
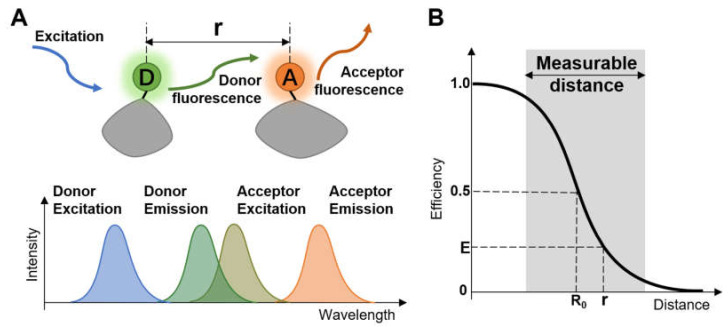
(**A**) Diagrammatic sketch of the concept of Förster resonance energy transfer; (**B**) Correlation between FRET efficiency and the distance.

Förster resonance energy transfer (FRET) describes the physical phenomenon of energy transfer between two photosensitive molecules, rendering convenience for real-time dynamic research of molecules under various physiological conditions [1,2,3,4]. FRET was first proposed in 1948 by Theodor Förster [5]. In FRET, energy from the excited donor chromophore may transfer to an acceptor chromophore in proximity through nonradioactive dipole–dipole coupling (Figure 1A) when the emission spectra of the donor and the absorption spectra of the acceptor overlap to some extent. Besides the overlap of spectra, the donor should have a sufficiently durative fluorescence lifetime and sit close enough to the acceptor to permit energy transfer to occur. So it is necessary to find a suitable donor–acceptor pair to satisfy the harsh conditions for effective energy transfer.

(1) $$E=\frac{1}{1+{\left(r/R_{0}\right)}^{6}}$$

FRET efficiency (E) is inversely proportional to the sixth power of the distance (r) separating donor and acceptor [6], expressed as the formula below:

where *R*_0_ is the distance when the energy transfer efficiency is 50%, called Förster distance (see Figure 1B), depending on the refractive index of the solution, the overlap integral of the donor emission spectrum with the acceptor absorption spectrum and their relative dipole moment orientation. Typically, the distance between the chromophore pair can be well distinguished at the range of 1–10 nanometers, making FRET efficiency extremely sensitive to small changes of distance. Therefore, FRET is a sensitive tool to obtain structural information of macromolecules and determine the approach between two molecules within several nanometers.

FRET is referred to as an effective “spectroscopic ruler” due to the above characteristics. Since FRET is not mediated by photon and the acceptor chromophore does not necessarily emit fluorescence, several methods have served as options for FRET detection. One of the most common methods is to measure the increase of acceptor emission because of the energy transfer from the donor [7]. Similarly, FRET efficiency can also be inferred from the donor fluorescence changes [8]. Another strategy is to monitor the photobleaching rates of the donor in the presence and absence of an acceptor, as the acceptor competes with the photobleaching pathways [9,10].

A common application of FRET is the measurement of distances between different regions tagged by chromophores of a single biomacromolecule, which provides its structural information and can be used to track conformational changes [11,12]. This use allows the dynamic study of protein–protein interaction under various physiological conditions, including detection of enzyme activity changes [13,14], movement and fusion of biomembranes [15,16], signaling transduction [17,18,19], to name just a few. Generally, these traditional FRET approaches measure efficiency on average in the bulk sample. The components to be considered are the two populations of donor and acceptor that interact to produce these signals rather than a pair of single chromophores. For some homologous populations having different FRET values, only a weighted averaged FRET value is obtained to represent them as a whole, thus unable to distinguish these populations [20].

With the ongoing progress of molecular biology research, it is becoming more and more important to analyze the behavior of a single molecule. Single-molecule techniques reveal the function mechanisms, conformation dynamics of individual biomolecules [21], which are usually heterogeneous and difficult to explore by ensemble-averaged methods. In contrast to ensemble FRET, single-molecule FRET (smFRET) provides signals of lots of parallel individual molecules wherein the single pair of donor and acceptor is exited and detected, which allows more precise analysis of heterogeneous populations. Specific FRET values of each molecule are accessible in smFRET instead of the average value overall. smFRET is able to monitor the short-lived populations in transition states [22] that are hard to characterize by ensemble FRET because they do not accumulate. Moreover, by tracking specific molecules, smFRET can measure system changes in equilibrium [23].

smFRET is usually used to identify the subtle structural differences of biomacromolecules dynamically and statically, especially for proteins. These changes are generally closely related to proteins’ folding pathway and functions. Some typical examples, including receptor and antigen interactions [24,25], vesical fusion [26], and ion channel dynamics, also relate to the conformational changes and equilibrium properties of molecules [27]. smFRET technologies have been put to use to study the folding dynamics of nucleic acids [28,29,30]. A single-stranded region in nucleic acids is likely to fold intramolecularly upon itself to form hairpins, internal loops, bulges, and junctions. Sequences rich in guanosine or cytosine can construct four-stranded structures, such as G-quadruplexes and i-motifs. These structures are formed to achieve functionality, like regulation of gene expression or disease process, and carry crucial genetic information not presented in genomic sequences. There is a large variety of studies by smFRET assays investigating the formation conditions and interaction mechanism of the secondary structure of nucleic acids, such as the hairpin ribozyme [31,32], Holliday junction [33], three-way junctions [34] and quadruplex [35,36,37].

In this review, we briefly summarize some representative novel applications of smFRET on investigating key biostructures and interactions. Methods and critical notes on comprehensively understanding the complexity of various biomolecules with smFRET are concluded and discussed. We hope this review can exhibit the versatility of smFRET and serve as a guide for the establishment of smFRET platforms for targeted objects.

## 2. Setting a Single-Molecule FRET Measurement

### 2.1. Imaging Strategies for smFRET

#### 2.1.1. Confocal Microscopy

The distribution of various conformational states of molecules is routinely done by counting free-diffusing molecules in solution. This approach is suitable for researching the distribution of proteins with different sites and does not demand long-time tracking of molecules, since they would not stay in sight for long. Although the concentration is not too highly controlled to inhibit observation, there are still a vast number of molecules randomly distributing in the solution and continuously moving into the monitored volume. Thus, the throughput of this strategy is typically high, allowing to obtain refined distribution of various molecule conformations for summarization or screening within a wide range of environmental changes or heterogeneous groups.

**Figure 2:**
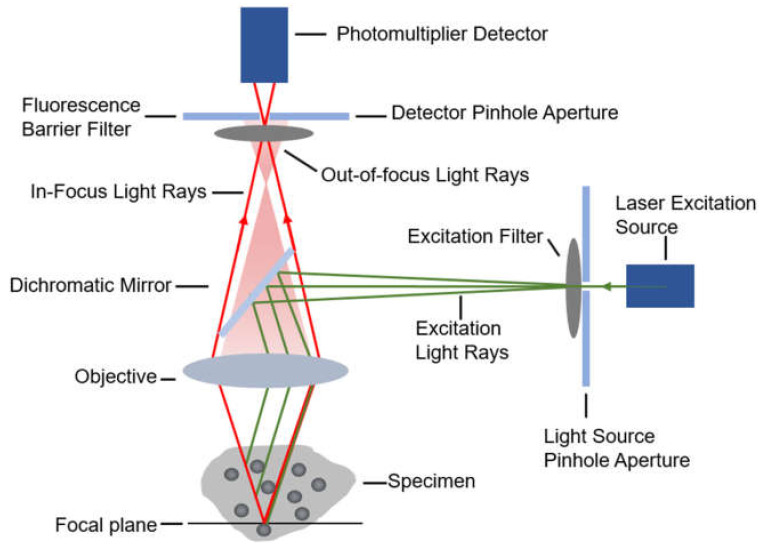
The diagrammatic sketch of the confocal microscope’s principle.

When diffusing single molecules labeled with fluorophores in a solution traverse the laser excitation volume, emission fluorescence is generated with a spatial amplitude enabling smFRET measurements. Using a confocal microscope to measure smFRET values of freely diffusing biomolecules (Figure 2) is relatively straightforward without immobilization-induced influences that may occur on the surface of immobilized molecules [38]. Confocal microscopy increases optical resolution by means of using point illumination and a pinhole to block out-of-focus signal in image formation. Coherent light emitted by the excitation source passes through the illumination pinhole placed behind the excitation source and is reflected by a dichromatic mirror, then focuses on a point of the focal plane of the specimen. Secondary fluorescence emitted by the point passes back through the dichromatic mirror and then focuses on the detection pinhole in front of the detector. Most of the extraneous light is blocked out, eliminating or minimizing background noise. Under the control of the computer, confocal microscopy can scan different layers in the specimen continuously and thus obtain a series of thin optical sections. Because a large number of events occur in a relatively short time, these data can be recorded and accumulate in order to build a distribution of molecular properties. Confocal microscopes have been utilized in many single-molecule studies, such as the denaturation of biomolecules [38,39], distinguishing subpopulations of heterogeneous analyte molecules [40,41], determination of the shape or dimensions of virus particles [42,43].

#### 2.1.2. Total Internal Reflection Fluorescence Microscopy

**Figure 3:**
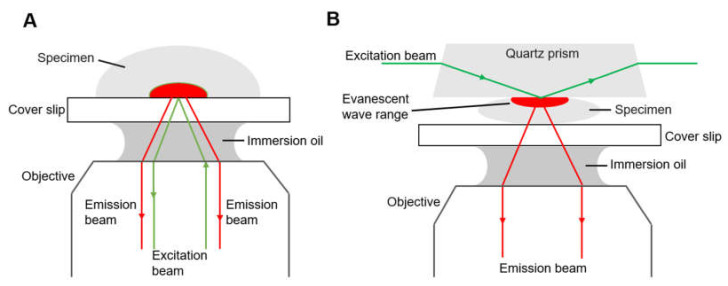
Diagrams of two types of total internal reflection fluorescence microscope, (**A**) objective-type TIRFM and (**B**) prism-based TIRFM.3.

Another type is total internal reflection fluorescence microscope (TIRFM, Figure 3). Total internal reflection is an optical phenomenon. If the incidence angle is greater than the critical angle, there is no refracted ray and the partial reflection becomes total when the light from an optically denser medium moves into an optically rarer medium. The fluorophores in a restricted region of the specimen are excited by the evanescent wave generated on the other side of the medium due to total reflection. The evanescent wave decays exponentially from the interface, and thus the detection depth within the specimen is approximately 100 nm, which reduces background fluorescence and increases the signal-to-noise ratio.

There are generally two types of total internal reflection fluorescence microscope, objective-based (Figure 3A) and prism-based (Figure 3B), having different optical paths. For the objective-type TIRFM, the excitation beam and the emission beam of the specimen are on the same side, and they pass through the same objective, collecting the maximum of fluorescence. The specimen is fully accessible, compatible with living cell treatment and open perfusion chamber [44]. However, stray light originating from the objective, dichroic mirror and other optics inside the microscope contaminate the emission signal. In the case of prism-based TIRFM, the emission beam and excitation beam are separated, and the fluorescence is collected by the objective on the same side of the emission beam, reducing the interference of the excitation beam. The prism-based system is easier to set up and the incident angle can be large so that the evanescent field is consequently thin. The evanescent field is generated on the opposite side of the objective; therefore, prism-based TIRF is not suitable for a thick specimen such as tissue sections. For a specimen in a closed flow cell, prism-based TIRFM is a proper choice, but an open perfusion chamber is easier to arrange with an upright microscopy. However, the prism-based TIRFM is the more commonly used for smFRET due to its superior signal-to-background ratio. Some studies of different research groups have described a novel, waveguide-based TIRFM system where the excitation beam propagates via the cover slip, with a prerequisite of larger optical power to obtain an equal intensity of the evanescent wave [45,46].

Compared with confocal microscopy, TIRFM does not image by scanning, which greatly improves the imaging speed, and the S/N ratio is better. Because of its imaging characteristics, TIRFM is suitable for long-time dynamic analyzing of molecules immobilized on a solid surface, which eases the observation while reducing the interaction between analytes. Note that additional biochemical passivation is needed, since the unfavorable interaction of fixed molecules with the surface may exert perturbations on the molecular biological activity and must be prevented. Molecules of interest can be immobilized through biotin-BSA [47], streptavidin in a deposited lipid bilayer [48], biotinylated poly (ethylene glycol) (PEG) [49,50] or using click chemistry [51]. When involving proteins, sometimes lipid vesicles are applied to encapsulate fluorescently labeled protein [52].

### 2.2. Fluorescent Labeling

#### 2.2.1. Choices of Fluorophore Pairs

As the principle of FRET suggests, the emission of the donor should overlap with the excitation of the acceptor to complete the process of transmission. Some sets of fluorescent pairs have been summarized with the accumulated experience from researches. Cyanine, Atto and Alexa series are the most commonly used fluorophores and are easily available from distributors. These small fluorescent groups can be modified on expected sites of biomolecules via covalent bonding and have low steric hindrance on molecules. With their relatively long photobleaching lifetime (more than a hundredth of a second) and quantum yield [53], these conventional dyes are more suitable for in vitro single-molecule FRET than fluorescent proteins, which have been widely used in non-single-molecule FRET due to their high bio-compatibility and specificity [54]. The organic fluorophores can also be used in living cells with proper modifications to detect single molecules in vivo [55,56]. Quantum dots (QDs) is another choice for smFRET labeling for their high brightness, photostability and adjustability. However, the physical size and the potential defect on the surface may introduce problems in practice. A recent review [57] detailed the features of QDs applications on smFRET comprehensively.

**Figure 4:**
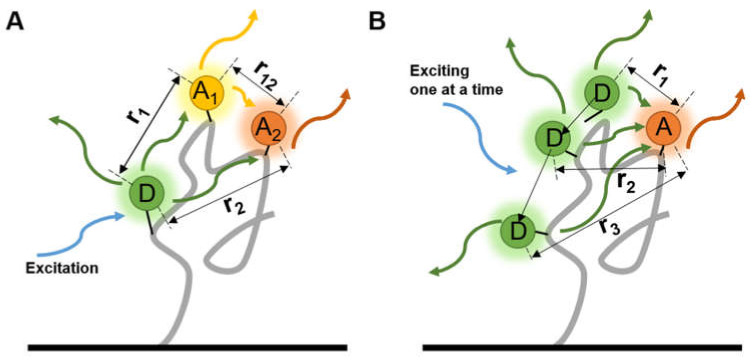
Two types of multi-labeled smFRET. Each molecule was labeled by (**A**) one donor and two different accepters at the same time, or (**B**) altering the loci of donor, to measure the distances among three or more sites.

Multiple FRET pairs provide more distance information among several known sites on a molecule than a single pair so the precision can be improved. A three-color smFRET, with one donor and two different accepters on each protein at the same time, was used to investigate the folding dynamics of proteins [58] and the influence between accepters was quantified (Figure 4A). The fluorescence lifetimes and the correlations of fluorophores can be improved with a high temporal resolution, and comprehensive exhibition of the bioprocess can be obtained through the complex labeling strategies. By detecting several pairs of fluorophores in a nucleic acid complex , Filius et al. [59] proposed “FRET X”, in which the detection of smFRET was sequentially done by hybridizing and exciting one donor-contained primer at a time (Figure 4B), and the dwell time was prolonged. The multiple labeling points and sequential signal detecting improved the accuracy of structure determination and can be coupled with the multiple virtual barcodes, developed by the coworker Kim et al. [60] to observe multiple orthogonal probes in a short time.

#### 2.2.2. Labeling with Low Impact

FRET requires at least a pair of fluorophores to enable the energy-transferring process from the donor to acceptor. Apart from the basic rules on distance and spectrum property of the fluorophores, consideration should be taken not to disturb the natural process of molecules with labeling groups while maintaining the detecting efficiency. While novel fluorophore pairs with high efficiency or robustness have been increasingly proposed, picking a labeling site is often a somewhat empirical work.

As a chain-like molecule, nucleic acid can be modified with fluorophores either on its bases or on terminal ends (i.e., 5′ end or 3′ end). Hartmann et al. [61] discussed the difference of these two strategies on a DNA self-loop test. The donor was placed on the complementary strand on the distal stem while the acceptor was fixed on the 3′ end or on the last base of the proximal stem to exhibit whether one complex formed a hairpin structure. Similar FRET efficiency and Gibbs free energy were shown in these two labeling strategies, indicating the molecular properties of open or closed remained the same regardless of the labeling method. Nevertheless, dynamic analyses exhibited a huge difference of transition-state free energies of the two types of modified DNA, suggesting a modified base can impact the natural function. However, labeling on bases could benefit the research in some particular cases. For example, G-quadruplexes (G4) can appear at the end or in the middle of a nucleic acid strand. Internal base labeling of guanosines [62] is compatible with the two types of G4 folding but the design should be optimized and verified to not impede the natural secondary structure [63].

Site-picking can be more difficult for protein labeling since the structure will be less orderly than nucleic acid. The site should be solvent accessible, unrelated to active sites while satisfying the distance requirement. Conflicting results on protein behavior were concluded [64,65,66] without a satisfying explanation until a corrective work emerged [67] where the labeling itself caused an unexpected change of molecules. A program-based site-picking method [68] was published recently, which can be helpful in the design.

### 2.3. Refined Structure Determination via smFRET

Although techniques such as cryo-EM and X-ray crystallography can obtain the structure of a sample with resolution up to atomic level, FRET still has its own place in analyzing molecular structure. Some structural features of the sample would change or be selected with bias during the crystallization step [69]. Also, the relatively high throughput and mild detecting environment make FRET able to easily screen the conformational heterogeneity of a vast number of molecules with time scale.

As an optical method, conventional smFRET has a limited spatial resolution due to the diffraction. Szalai et al. [70] successfully realized sub-diffraction imaging by migrating the STED technology to smFRET. Labeled antibodies were used to bind the surface of neuron cells, and images with sub-diffraction resolution were obtained. Molecular interactions within diffraction range and structure distinction based on multi-color labeling will be significantly improved as optical technology is developed.

The state transition of molecules is not expected to be too fast for the sake of observation. When the transition time is short, then the exposure time (typically from macro to milliseconds) detected signals may contain time-averaged data. Fluorescence correlation spectroscopy (FCS) combined with smFRET can quantify signal dynamics down to the nanosecond [71] and the combination of FCS and FRET would increase the temporal limit of the latter. Internal friction in different transition states of domains was quantified at sub-microsecond scale and its distribution of the specific regions along the free-energy surface described [72].

## 3. Investigating the Structural Changes under Various Circumstances

### 3.1. Screening Conformational Changes of Proteins

The structures of proteins are not absolutely stable. On the contrary, they transform according to external environments. In particular, intrinsically disordered proteins (IDPs) are proteins that do not have a stable folding structure, and the peptide chains show conformational flexibility in solution. With proper labeling pairs, the dynamic of conformational changes, occurring partially or totally, can be observed in terms of the distances between marked sites that are typically located between 2 nm and 10 nm. The FRET efficiency of many individual molecules has been calculated and the extent of folding obtained according to the histogram of the signal. Consequently, heterogeneous structures were divided and the abundance change was tracked through time ranging from microseconds to seconds [73]. The strategy of sample dispersing (i.e., diffusing in solution or tethered on substrates) is chosen based on the molecular characters for various purposes and care should be taken respectively on experimental design.

**Figure 5:**
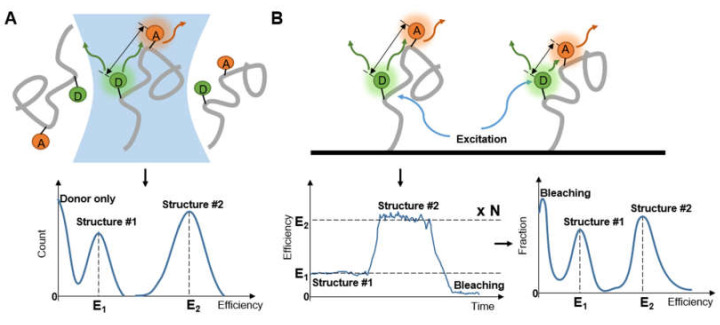
Observing (**A**) diffusing molecules and (**B**) immobilized molecules to obtain distribution of molecular structure.

A high throughput test of a series of diffusing molecules (Figure 5A) provides a comprehensive view of the relationship between molecule conformation and the external environment, by which the systematical knowledge of molecular reactions, diseases or life form can be accumulated step by step. The multi-stage unfolding of small globular protein under surfactant solution was systematically investigated [74]. Complexity of SDS-mediated unfolding of protein was described under SDS concentration of 0–300 mM and was divided into several parts with the transition rate. Heterogeneity of heat shock protein (Hsp) and Hsp40-modulated Hsp70 chaperone cycle were investigated on a sub-second timescale in detail [75]. smFRET of complexes under different external environments exhibits the process of undocking and dimerization, with the alteration of ATP lysis ability. Despite its high throughput, the strategy of counting dispersed molecules is mostly applied at the temporal scale of milliseconds, long-time conformational change may not be observed completely if the object moves out of sight.

Transition time is not the primary parameter to consider in the measurement of immobilized molecules (Figure 5B), but care should be taken to verify that the nature of the molecule is not impacted by immobilization. It was stated early on that posttranslational modifications are associated with IDPs [76], and the known relationship of modification and intrinsic disorder was summarized [77]. Choi et al. [78] tracked immobilized C-terminal domain (CTD) of the GluN2B subunit of N-methyl-D-aspartate receptor (NMDAR) for over 40 s with 100 ms per frame to find differences after Src phosphorylation. The conformational change was observed without the obvious appearance of interaction between molecules. Apart from a site that was too short (15 residues) to exhibit the change, seven out of the eight paired sites were confirmed to be expended but not as much as a random coil after phosphorylation. With a BSA-passivated surface, comparisons were done to show that the influence of surface tethering on natural conformational changes did not alter the transition trend. Interestingly, the extent of measured change was amplified by the bonding strategy.

Single-molecule monitoring usually requires dilution steps to decrease the concentration of analytes so that there are few crowds to disturb the observation. However, the low input concentration may lower the signal-to-noise level or even impact the natural process of bioreactions. This restriction can be widened when preparing immobilized molecules because discrete chambers that impede the formation of large crowds are achievable on a solid substrate. The parallel small chambers of zero-mode waveguide (ZMW) were utilized to study the binding events of fluorescently labeled cyclic guanosine monophosphate (fcGMP) to monomeric cyclic nucleotide-binding domains (CNBDs), and the concentration limits were pushed up to over 100-fold [79]. The conformational dynamics of single molecules were traced for tens of seconds, and the dwell time showed clear patterns with prolonged bleaching time at high concentration. Long-time conformational change detection via smFRET also has widespread applications in describing nucleic-acid-involved reactions, which will be discussed afterwards.

Other technologies such as small angle X-ray scattering (SAXS) and nuclear magnetic resonance (NMR) have been widely combined with smFRET to comprehensively understand and predict the conformational changes. Gomes et al. [80] exhibited the consistency of these three most common tools in describing the conformational ensembles of Sic1, pSic1 and suggested integrating data for comprehensive knowledge of complex molecules.

### 3.2. Dynamic Changes in Nucleic Acid Structure

Nucleic acid stands (i.e., DNA and RNA) have linear structures, and the principle of hybridization is based on the well-known complementary base-pairing. Consequently, the distance change between sites, which is induced by extension, annealing, compaction or secondary structures of strands, can be more predictable than that of proteins, and fluorescent labeling on nucleic acid molecules has been routinely used in the smFRET measurement of structures and assemblies. Based on the widely used varieties of modification methods, these strands can be conveniently tethered upon solid substrates and labeled on either terminal ends or specified intermediate units, giving researchers sufficient freedom to design the protocols.

#### 3.2.1. Non-Helix Secondary Structures of DNA

**Figure 6:**
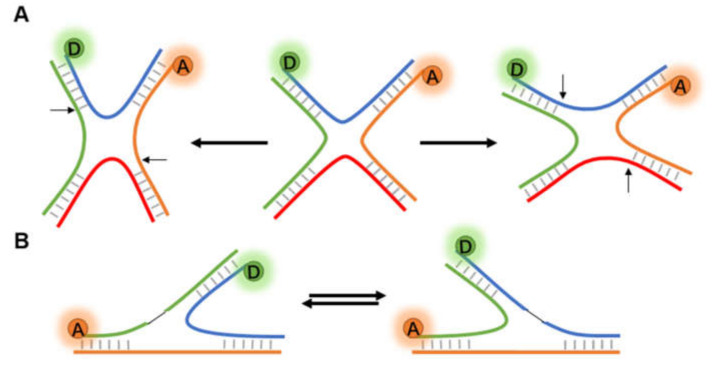
Structural change of Holliday junctions. (**A**) The diastolic four-way Holliday junction changes to either potentially compact X structures in the presence of cations or other accelerant. (**B**) Two different conformational isomers of three-way junction.

The fundamental function of DNA is as the heritable carrier of biological genetic information. Direct observation of the dynamic conformational changes of DNA is helpful to understand the action mechanism of nucleic acid [23]. Holliday junction (HJ), a central intermediate in DNA repair and homologous recombination (HR), is a branch structure connecting two DNA double strands [81,82]. HJ usually exists in solution as a four-way X-stacked conformer, exhibiting heterogeneity in crossing and continuous state transitions, and has been extensively studied by FRET [33,83,84] (Figure 6A). In the late stage of HR, HJ is broken down into normal double-stranded DNA (dsDNA) by an enzyme system called RuvABC [85,86].

Gibbs et al. [87] conducted smFRET experiments in different ionic environments (Mg^2+^ and Na^+^) to investigate the influence of RuvA-HJ interaction on the conformation of the HJ. The experimental results indicated that RuvA bounded to the HJ stably through electrostatic interaction to prevent its conformational dynamics. Recently, a more detailed report [88] not only focused on the binding properties of RuvC and HJ, but also dissected the behavior of the HJ-RuvC complex to continue cleavage after recognizing the cleavage-active sequence. They discovered that RuvC can achieve site-specific cleavage, which may inspire further research using this mechanism of RuvC. Similarly, GEN1, a cytoplasmic homologous recombination protein, is considered as one of the key factors in resolving the persistent HJ after the dissolution of the nuclear membrane [88,89,90]. Based on previous studies, Sobhy et al. [91] combined smFRET with other techniques to explain the kinetic details of how GEN1 dimers decomposed HJ.

In addition to the four-way HJ (4WHJ), smFRET has also been applied in determining the conformational dynamics of three-way junction (3WJ, Figure 6B). Leveille et al. [92] first reported the application of smFRET in the helical stacking arrangement for a series of bulged-in DNA 3WJs. Moreover, replication slippage is a frequent reason for the emergence of 3WJs. Hu et al. [93] designed 3WJs with slip-outs of between 2 and 30 CTG or CAG repeats, and the FRET signals showed that repeats of slip-out led to a two-state behavior. Based on previous works and existing data, the authors proposed a model of reversible branch migration in mobile 3WJs with trinucleotide repeats, which may help the treatment of diseases.

#### 3.2.2. Secondary Structures of RNA

Similarly, deciphering the mechanism of how RNA folds to complex structures is of great significance for recognizing its functional characteristics. The monovalent and divalent cations play a pivotal role in promoting RNA conformational transformation. For instance, monovalent cations (e.g., K^+^, Na^+^) facilitate the first step of RNA folding to secondary structures, followed by divalent cations (e.g., Mg^2+^) that further enhance secondary structure interactions and tertiary contacts [28,94]. Previous studies in the role of metal cations in RNA folding and kinetics have concentrated on secondary structure formation (e.g., 3WJs [34,95], kissing hairpins [96], GAAA tetraloop-receptors [97], four-way junctions [98], catalytic ribozyme folding [99], RNA/RNA or RNA/DNA interactions [100]). The docking of GAAA tetraloops with specific receptor sequences forms extensive and abundant tertiary motifs in RNA. Bisaria et al. [101] selected the extensively studied P4–P6 domains of *Tetrahymena* ribozyme and smFRET to provide evidence for the specific binding of Na^+^ and K^+^ to RNA tetraloop–tetraloop receptor (TL-TLR) tertiary motif. Furthermore, Sengupta et al. sequentially studied the kinetics of TL-TLR folding–unfolding in amino acid environments (lysine, arginine and glycine) [102,103]. They proved that arginine and lysine interact with nucleic acids in a manner similar to monovalent cations. Interestingly, D- and L-arginine have strong chirality dependence on the inhibition of TL-TLR folding, but it is not certain whether any other selected chiral species have similar chiral sensitivity characteristics. These works reflect the value of junction sequences and interactions with cations in RNA folding. It should be noticed that organic cosolute can also significantly affect the conformational transformation of nucleic acids except cations. Holmstrom et al. [104] chose high-solubility small molecules, trimethylamine N-oxide (TMAO) and urea to understand the substantial effect of these substances on the conformational transformation of nucleic acids. According to smFRET data analysis, it was concluded that the nucleic acid folding could be altered by osmotic pressure, which is a pioneering experience. smFRET plays an essential role in the investigation of conformational dynamics of nucleic acids at the single-molecule level due to its convenience, high resolution and scalability.

### 3.3. Complex Chromosome Structures

**Figure 7:**
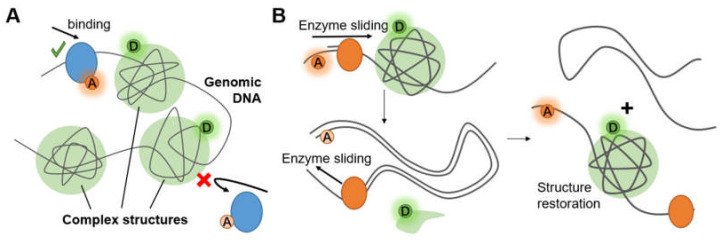
(**A**) Complex structures on chromosomes alter accessibility. (**B**) Complex structure restored after being destroyed by enzyme sliding.

Long genomic DNA in eukaryotic cells has been compacted to form chromosome structures. These structures protect the fragile nucleic acid by bonding proteins (e.g., histones) or hiding them behind certain non-coding sequence (e.g., G-quadruplexes). Accessibility of gene (Figure 7A) and conformational change of the structures (Figure 7B) are two of the most researched hotspots that exhibit the principle and efficiency of compaction and protection.

#### 3.3.1. G-Quadruplex of Telomeres

G-quadruplexes (G4) are structures formed by guanine-rich areas and were named after the π–π interactions among the stacks, each of which contains four guanine bases. The principle of FRET has been applied to distinguishing inter- and intramolecular G4 in bulk solution since 2002 [105] and is still an efficient way to test the formation process of G4 in various conditions [106,107].

The appearance of G4 structures at the end of chromosomes, namely telomeres, can protect the inner DNA from degradation. Conformers of G4 have been divided based on the direction of strand. Long et al. [108] investigated the state of G4 in various ionic environments. The donor was labeled on the intermediate primer while the acceptor was at the free end of the G4-containing strand. The distance between the free end and primer changed according to the direction of the G4-forming strand, so the formation of the captured molecule could be decided. Three different conformers of G4 were observed and their distribution under KCl treatment of several levels were concluded to guide further designing of G4 telomere analyses. Recently, varieties of G4-terminal DNA strands were immobilized to trace their dynamics within minutes [109]. An acceptor-labeled PNA probe repeatedly annealed with the intermediate area of G4 strand. The dell time and frequency were evidence that the accessibility of telomeres varies, confirming that long telomeres can protect strands from small molecules or nuclease better than short ones with their compact conformation.

smFRET exhibited not only the structural features but also the process of destruction of telomeres [110]. Fluorophores were labeled at each side of G4 structures in pairs to monitor the disruption of parallel G4 by telomerase translocation. With the efficiency drop of smFRET, the character signal of the dynamic process of enzyme-induced G4 unfolding was extracted. This research pointed out the mechanism of RNA-templated G4 extending by human telomerase and the failure of ligand protection of telomeres. From another aspect, Parks et al. [111] analyzed the RNA structural rearrangements in the telomerase complex with smFRET and FRET-guided modeling. The two works on telomere also applied a simulating method to further prove the molecular principle based on the observation of smFRET. The combination of dynamical and structural analyses with model simulation completed the realization of the bio process at single-molecule level.

#### 3.3.2. Histone–DNA Complex of Nucleosomes

DNA in chromatin is compacted in the form of nucleosome, in which genomic DNA is wrapped around small protein assemblies called core histones and coiled structures are lined together to form higher-order structures by linker histones [112]. One single nucleosome can include ~50 nm double-strand DNA molecules that wrap around the histone core and may slide or even come off spontaneously (by DNA breathing) [113].

The intra-nucleosome dynamics was observed through smFRET as early as 2005 [114]. A nucleosome-positioning element was amplified via PCR, of which one primer was labeled with the acceptor and the other set was labeled with the donor, so the amplicon (164 bp) contained a whole pair of fluorophores with a certain distance (25 nm), and the immobilized DNA compacted with histone was expected to show a high FRET efficiency with the distance decreasing to ~3 nm. The nucleosome that formed under high salt concentration could survive various environments with a greater possibility of staying closed under higher salt concentrations, which may be attributed to the stiffer DNA structure. The different states of the opening chromosome were also discussed. This primer-labeling method has been widely used to determine the conformational dynamics of single nucleosomes [115,116,117]. Coupled with advanced techniques, the transitions in nucleosomes were described in detail, and the sequential scheme of nucleosome assembly and disassembly was drawn at the scale of histone oligomers [118,119].

The fluctuation of histone–DNA compaction is not always random and can be connected with ongoing bioreactions. The opening of the nucleosome was observed to be a spontaneous step-by-step model, instead of a behavior forced by polymerase, when they were giving way to nucleic acid extension [119]. The results showed that most assemblies became completely disassembled after the elongation, but the principle of re-assembling remains uncertain. In the process of DNA damage response, chromatins were regulated de-compacting at the laser-induced breakpoint to ease the repairing while compacted chromatins gathered around the repair locus demarcating the lesion [120]. Remarkably, this work labeled nucleic chromatins inside live HeLa cells and observed the interaction in situ with FRET, providing a novel platform for further chromatin biology.

Representative smFRET studies on the structural change of biomolecules under various conditions were summarized (Table 1) according to the type of sample and variables in measurements.

## 4. Tracking the Interactions between Biomolecules

### 4.1. Virus Spike–Host Interaction

Virus spike on the surface of virus envelope is generally composed of glycoproteins and is essential for virus infection. They mediate the attachment of virus to the host cell and promote the fusion of virus envelope and cell membrane [121]. The virus undergoes some conformational rearrangements to interact with host cells, hence, understanding the structural dynamics of virus spike is of great significance for vaccine interventions and anti-viral treatments.

smFRET can exhibit the dynamic nature of the virus spikes labeled with fluorophores. Since the COVID-19 epidemic, smFRET has been used to study its spike, S-glycoprotein, which is made up of three smaller protein subunits [122,123]. Each subunit has two domains, S1 and S2. The S1 domain recognizes and binds to human angiotensin-converting enzyme 2 (hACE2), the protein on the human cell membrane, which initiates the structural changes of the S2 domain to help virus entry. By introducing donor and acceptor fluorophores before and after the receptor-binding motif on S-glycoprotein, smFRET revealed at least four distinct conformational states between the closed ground state and the open state, reflected by different levels of FRET values [124]. This investigation also verified that the binding of the hACE2 receptor converts S-glycoprotein conformation to low FRET (~0.1), manifested as receptor-binding domain up conformation [124], which provides the basis for vaccine development.

**Figure 8:**
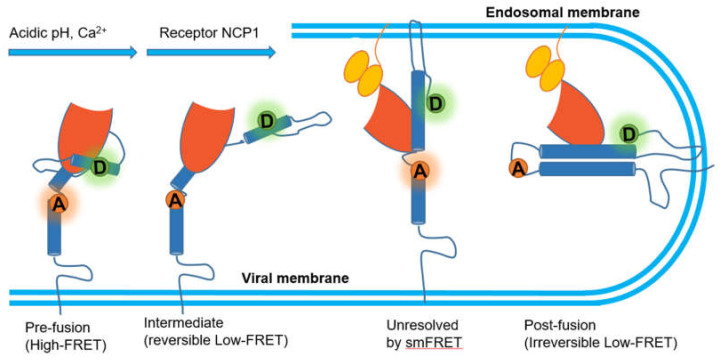
Mechanistic model of GP-mediated membrane fusion.

Ebola, another deadly virus, can cause a viral hemorrhagic fever in humans and other primates. Its envelope glycoprotein (GP) is composed of trimers, each with GP1–GP2 heterodimers of which GP1 is responsible for the attachment to cells while GP2 promotes the membrane fusion [125,126]. A series of independent studies have presented evidence supporting that Ebola virus needs binding to human Niemann–Pick C1 (NPC1) protein to enter human cells [127,128]. By labeling fluorophores at two position of GP2, one at the N-terminus and the other proximal to the fusion loop, Das et al. developed a smFRET imaging assay to observe the conformational transition of GP, where acidic pH and Ca^2+^ shift the conformational equilibrium of GP in favor of a conformation optimal for NPC1 binding with low FRET value [129]. However, the authors pointed out that there were two different conformations behind the low FRET. In particular, they restored neutral pH or removed Ca^2+^ and found out the high FRET conformation before fusion was reformed, which indicated that the process mediated by acidic pH and Ca^2+^ was reversible. In contrast, NPC1 binding then facilitated GP into another irreversible low FRET conformation that might be the post fusion conformation 6-helix bundle (6HB) or the transition state preceding 6HB (Figure 8). In this study, the external conditions were altered to reveal these two critical transitional states, though they were inseparable in the FRET spectrum.

### 4.2. Dynamics of Membrane Proteins Folding

Membrane proteins interact with lipid membranes and varying environments in order to perform various functions, including substance transportation, ion homeostasis, signal transduction, catalysis, and other specific functions. Almost all function implementations involve multiple coexisting and mutable conformations. FRET on a single-molecule level is suitable for studying membrane-protein conformational changes, owing to the advantage of accessing heterogeneities and identifying the subpopulations in the complex conformation set. Furthermore, smFRET allows investigations of membrane proteins under lipid bilayers or membrane mimetic environments, precluding interfering with lipid bilayers or their mimics in ensemble methods [130,131].

Yano et al. [132] investigated the association–dissociation dynamics of a simple de novo-designed transmembrane helix (AALALAA)_3_ in a model lipid membrane, focusing on the nonspecific effect of cholesterol. The helices preferentially formed antiparallel dimers that were monitored by attaching Cy3B (donor) and Cy5 (acceptor) at each helice (III and IV), respectively. This FRET pair can detect both parallel and antiparallel dimers due to the relatively long critical transfer distance. They confirmed that cholesterol significantly stabilized the antiparallel helix dimer interpreted as hydrophobic interaction. In another study two years later, they replaced the center of (AALALAA)_3_ with the guest GXXXG motif [133]. Interestingly, transient dimerizations in both parallel and antiparallel topologies achieved an equilibrium, whereas the addition of cholesterol completely abolished the Gly-mediated associations. They suggested that the reason could be the smaller crossing angles and enhanced flexibility of the dimers on account of the introduction of Gly residues, while more definite evidence has yet to be found. On the aspects of membrane proteins, other similar studies have provided a basic insight on various conformation landscapes, including membrane-protein misfolding [134] and structural dynamics of the ion channel [135]. Other tools like fluorescence correlation spectroscopy (FCS) [136,137], super-resolution microscopy [138] and infrared spectroscopy [132,133] are combined with smFRET for better analysis of the mechanism behind the phenomenon.

### 4.3. Protein Aggregation

Protein aggregation formed under certain circumstances has been widely found in many neurodegenerative diseases [139]. The high throughput character of distance analysis of smFRET empowered screening on aggregation-prone structures and conditions of proteins [140].

Polyphosphate in solution can act as an aggregation promotor of tau [141]. smFRET exhibited the extent of polyphosphate-induced impacts on tau, in which the structure was compacted and the long-range interactions were disrupted. Binding efficiently with multiple sites of tau, polyphosphate enhances aggregation through the intermolecular cross-linking. The aggregation-prone structures and their aggregating path in the present of tubulin [142], heparin and sodium chloride [143] have been studied to complete the knowledge of tau aggregation.

Instead of being paired labeled on same molecule, the donor and receptor of FRET can be bonded to different proteins to exhibit their aggregating process directly through the intermolecular distances. Klenerman et al. [144] incubated α-synuclein tagged with either AF488 or AF647 for various times and distinguished several different structures of oligomers by their unique FRET efficiency. The efficiency can indicate the size and level of compaction of the oligomers by revealing the average distance between neighboring tagged sites. This strategy could be integrated with microfluidics [145], providing even more comprehensive and detailed insight into oligomer formation under various conditions.

### 4.4. Synthesis of DNA Strands

The principle that new nucleic acid strands are template-synthesis-based is one of the most common universal rules of lives. The elongation of primer depends on the movement of polymerase along the strand so the distance between the enzyme and any fixed point on the template changes during the process. Based on that, smFRET measurement of DNA strand extension was done at base-pair resolution [146]. DNA elongation by Klenow fragment of DNA polymerase I (KF) was controlled by adding only the required dNTPs (N = T, A, G) and the different attitudes of the sites of single polymerase were observed to exhibit different levels of FRET. Repeated patterns indicated that KF moved forward to accomplish the addition of one dNTP to the primer after a rapid conformational fluctuation when recognizing a dNTP and three statuses corresponding to the polymerase, proof-reading and transient sites were distinguished. DNA elongation by various polymerases was visualized but the donor and accepter can be both labeled on the strands to minimize the impact of modification to the enzyme and improve its generalization [147]. This strategy was based on the difference in flexibility of single- and double-strand DNA since weak single strand will be stiffer as a double helix after the completion of strand synthesis. Although equipment requirements could be higher than the previous method, the processes of binding and sliding of polymerase on strands were clearly observed and the conformational fluctuation was further discussed.

In a more recent work, multiple functions of DNA polymerase I were investigated with designed DNA strands with levels of mistakes [148]. The interactions of the polymerase domain and/or the exonuclease domain were traced and distinguished with FRET efficiency regulated by the distance between known labeling sites on the enzyme and strand. The site of complex transited at various rates as the template having different types of error and the ratio of preferable structures could help to understand the nature of DNA polymerization and predict enzyme behavior. Further, parallel labeling of different domain-base pairs may improve the bandwidth of measurement, of which more conformational information can be obtained from a single experiment, to decrease the potential errors in the comparison among repeated same biological processes with different fluorescent labeled sites.

### 4.5. Imaging Molecular Behavior in Living Cells

Deciphering the various static and dynamic nature of molecules in the environment of living cells is the crux of understanding the complexity of cell functions. However, the movements of these molecules may be involved in stochastic processes and characterized by a wide temporal and spatial complexity [149]. smFRET is indispensable for intracellular researches of molecular detection and tracking due to its high sensitivity, specificity and non-invasiveness. The pivotal challenge in tracking a single molecule or multi molecules by smFRET in vivo is to produce signals at high noise background and extracting the necessary mechanical information [56].

**Figure 9:**
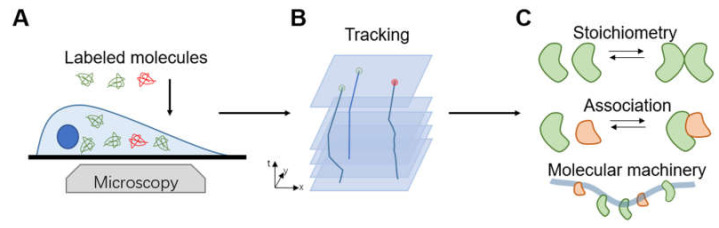
Work flow and applications of smFRET in single-molecule imaging in living cells. (**A**) The selection of appropriate fluorescent probe and dye pair is the primary critical step. Then the biomolecules are imaged using an optical fluorescence microscope with ultrahigh resolution and monomolecular sensitivity. (**B**) Single-molecule localization and tracking algorithms are employed to determine the position and motion state of the labeled molecule. (**C**) smFRET has been successfully applied in multifarious biomedical applications.

Figure 9 shows a typical work flow of molecular imaging utilizing smFRET in cells. Disorders and abnormalities in the Ras-Raf-MAPK system are involved in many types of human cancer [150,151]. In 2004, Murakoshi et al. [152] applied smFRET to observe changes in the activation state of the small G protein Ras at the single-molecule level. Yellow fluorescent protein (YFP) and fluorescent GTP analog BodipyTR-GTP were used as donor and acceptor, respectively. The detection of sensitized emission of BodipyTR-GTP indicated the state of Ras activation directly, which provides a powerful tool for the study of the transduction mechanisms of various G proteins or other signaling pathways. Later, Hibino et al. [153] and Okamoto et al. [154] studied the conformational state transition of Raf, a cytoplasmic serine/threonine kinase via smFRET, to further discuss how diverse conformations and subtypes affect Ras-Raf recognition. These reports further propelled the application of smFRET in studying signaling pathways in living cells.

Benefiting from the convenience and continuity of smFRET, studies have focused on the dimerization and modifications of growth factors and receptors involved in signal transduction [155,156]. For instance, Class C G protein-coupled receptors are the major targets of many drugs, which constitute the largest class of transmembrane (TM) protein receptors. Asher et al. [156] combined smFRET and fluorescence recovery after photobleaching (FRAP) to monitor the assembly and structural dynamics of several GPCRs in living cells. They provided a strategy for detecting and tracking of TM proteins diffusing within the plasma membrane of biological cells. In addition, appearance and dynamics of nucleic acid complexes were also observed in vivo. Antonio et al. [157] developed a G4-specific fluorescent probe (SiR-PyPDS) that enables real-time single-molecule monitoring of individual G4 structures in living cells. They revealed the dynamic formation of intracellular G4s based on smFRET and found the formation is cell-cycle-dependent. It is likewise destroyed by chemical inhibitors during replication and transcription, causing fluctuations between folded and unfolded states. Interestingly, the versatile FRET can be even utilized to kill cancer cells specifically [158].

Representative smFRET studies on the interactions among biomolecules were summarized (Table 2) according to the involved objects and bioprocesses.

## 5. Conclusions and Outlook

As a well-known molecule ruler, smFRET indicates the distance between labeled sites of molecules by the efficiency of energy transfer. smFRET has been widely applied in exploring the conformational dynamics and interactions of molecules at nanometer scale and is compatible with either heterogeneity statistics or dynamic tracking of single biomolecules.

The elegant principle of FRET keeps the experiment of smFRET simple, which has low requirements regarding specialized equipment or solvent environment. Consequently, a vast amount of works have contributed to study single-molecular conformational changes under different external conditions. However, the resolution of smFRET may not be as high as some non-real-time techniques. In addition, smFRET can only show the distances of labeled sites rather than of the entire structure of molecules. To comprehensively understand the dynamic structure change of complex molecules, it is recommended to use these highly precise methods to obtain the discrete conformations with fine resolution while coupling the smFRET of specified sites to exhibit the process of changing continuously or in more complex conditions.

smFRET has been routinely used in observing interactions, thanks to its continuity in measuring. The reaction-induced distance change can be monitored conveniently for a rather long period and no major change in solution is required for the measurement. However, the output (i.e., distance between sites) of smFRET again has its limitation on globally indicating bioreactions. smFRET data usually serve as evidence to validate a model based on current knowledge or simulation, so coupling with other methods is also a good choice in this field. Nonetheless, the flexible solution setting of smFRET makes it an irreplaceable tool to visualize various interactions in real time, and the application can be expanded to monitor interactions in living cells.

Molecular behavior is close to its natural state when there are few restrictions on solvent conditions during measurement, so smFRET with high compatibility has been widely used in indicating bimolecular dynamics. Coupled with modern technologies with higher temporal and spatial resolution, smFRET improves knowledge of the structure and interaction of biomolecules in various bioprocesses. With the evolution of material and detection technologies, smFRET will continue to be a promising tool for unveiling the delicate principle of nature.

## Figures and Tables

**Table 1:** Representative smFRET studies on the structural change of biomolecules.

Sample	Variables	Key Findings and Significances	Ref.
Proteins with various states	SDS concentration	Transition among several sites was described under different solutions within a large concentration range	[74]
	Posttranslational modification	Domains were immobilized to record the conformational change	[75,78]
	Protein regulation	Transition states of Hsp70 chaperone cycle was shown to associate with various activity levels	[75,78]
		The concentration of sample was increased largely with parallel small chambers of ZMW	[79]
DNA secondary structure	Solvent condition	Dynamics intrinsic to HJ were analyzed to find cognate sequence and achieve site-specific cleavage	[88]
		Kinetic details of GEN1 dimers decomposed HJ were explained	[91]
	Strand sequence	A model of reversible branch migration in mobile 3WJ with trinucleotide repeats was proposed, which may help the treatment of diseases	[93]
RNA secondary structure	Metal cation	Na^+^ and K^+^ were proved to facilitate the formation of RNA tetraloop–tetraloop receptor tertiary motif	[101]
	Small molecules in solution	Arginine and lysine interacted with nucleic acids in a manner similar to monovalent cations, and arginine had strong chirality dependence on the inhibition of TL-TLR folding	[102,103]
		TMAO and urea were demonstrated to alter the nucleic acid folding by osmotic pressure	[104]
Chromosome Structure	Strand Sequences	PNA–probe binding was detected repeatedly to evaluate the accessibility in the telomere area	[109]
	Presence of telomerase	Enzymatic destruction of telomere was described and computer simulation was coupled	[110,111]
	Solvent condition	Primer-labeling method used to determine the dynamics of single nucleosomes	[114,115,116,117]
	DNA damage	Chromatin function in DNA damage response was observed dynamically in living cells	[89,120]

**Table 2:** Representative smFRET studies on the interactions among biomolecules.

Interaction	Bioprocess	Key Findings and Significances	Ref.
Protein–Protein	Virus infection	smFRET imaging assay revealed structure arrangement of critical binding domains between the membrane receptor and virus spike	[124,129]
	Protein aggregation	Intramolecular FRET showed the states of aggregation in various conditions quantitatively	[141,142,143,144,145]
		Intermolecular FRET exhibited the aggregating principle	[144,145]
Protein–Lipid	Cross-membrane transport	smFRET with long critical transfer distance detected both parallel and antiparallel dimers of transmembrane helix regulated by cholesterol	[132,133]
Protein–Nucleic acid	DNA synthesis	Drops in FRET efficiency indicated DNA synthesis with label on polymerase or on template	[146,147]
		Various labeling strategies can present the states of different active domains on polymerase	[148]

## Data Availability

Data sharing not applicable.
